# The Alleviation of LPS-Induced Murine Acute Lung Injury by GSH-Mediated PEGylated Artesunate Prodrugs

**DOI:** 10.3389/fphar.2022.860492

**Published:** 2022-05-20

**Authors:** Dan-Li Hao, Ya-Jie Wang, Jia-Ying Yang, Ran Xie, Ling-Yu Jia, Jin-Tang Cheng, Hai Ma, Ji-Xiang Tian, Shan-Shan Guo, Ting Liu, Feng Sui, Yu Zhao, Yan-Jun Chen, Qing-He Zhao

**Affiliations:** Institute of Chinese Materia Medica, China Academy of Chinese Medical Sciences, Beijing, China

**Keywords:** acute lung injury, artesunate, drug delivery, lipopolysaccharides, micelles, PEGylation, prodrug

## Abstract

Acute lung injury (ALI) or its aggravated stage acute respiratory distress syndrome (ARDS) is a common severe clinical syndrome in intensive care unit, may lead to a life-threatening form of respiratory failure, resulting in high mortality up to 30–40% in most studies. Nanotechnology-mediated anti-inflammatory therapy is an emerging novel strategy for the treatment of ALI, has been demonstrated with unique advantages in solving the dilemma of ALI drug therapy. Artesunate (ART), a derivative of artemisinin, has been reported to have anti-inflammatory effects. Therefore, in the present study, we designed and synthesized PEGylated ART prodrugs and assessed whether ART prodrugs could attenuate lipopolysaccharide (LPS) induced ALI *in vitro* and *in vivo*. All treatment groups were conditioned with ART prodrugs 1 h before challenge with LPS. Significant increased inflammatory cytokines production and decreased GSH levels were observed in the LPS stimulated mouse macrophage cell line RAW264.7. Lung histopathological changes, lung W/D ratio, MPO activity and total neutrophil counts were increased in the LPS-induced murine model of ALI *via* nasal administration. However, these results can be reversed to some extent by treatment of ART prodrugs. The effectiveness of mPEG_2k_-SS-ART in inhibition of ALI induced by LPS was confirmed. In conclusion, our results demonstrated that the ART prodrugs could attenuate LPS-induced ALI effectively, and mPEG_2k_-SS-ART may serve as a novel strategy for treatment of inflammation induced lung injury.

## Introduction

Acute respiratory distress syndrome (ARDS) is a common syndrome of respiratory failure with high rates of morbidity and mortality, which is a cascading process developed from acute lung injury (ALI), regarded to be induced by noncardiogenic pulmonary edema and hypoxemia, which often requires for mechanical ventilation on critically ill patients (Matthay et al., 2019). The development of ARDS is commonly associated with indications such as bacterial and viral pneumonia, smoking or chemical induced inhalation injury, and nonpulmonary infections, including sepsis syndrome, severe trauma, shock, aspiration of gastric contents, as well as pancreatitis, drug reactions and fat embolismic (Rehberg et al., 2009; Sakai et al., 2014). Nearly 150,000 people were diagnosed with ARDS in the United States each year, and 75,000 deaths were reported in the US annually (Bellani et al., 2016; Butt et al., 2016). However, the treatment for this syndrome remains quite challenging despite of decades of efforts on intensive basic and clinical research (Ashbaugh et al., 1967; Huppert et al., 2019). Noting that in most cases about the progression of ALI to ARDS develop within 2–5 days of hospitalization, therefore, the interruption of the progression from ALI to ARDS is greatly preferred (Gajic et al., 2011; Ruthman and Festic, 2015). With increasing in-depth studies of ALI/ARDS, significant breakthroughs have been made (Shaw et al., 2019), however, none of them were effective in clinical trials, and the mortality rate remains high (He et al., 2021).

Acute lung injury (ALI) is a lung disease characterized by pulmonary edema caused by inflammatory imbalance and destruction of alveolar/capillary barrier (Ranieri et al., 2012). Inflammatory responses are the physiological reflection of the body to various pathological damages and stimuli. It is generally regarded that uncontrolled inflammation of the lungs or the whole body is the main pathogenesis of ALI/ARDS. During the process, cells including polymorphonuclear neutrophils (PMNs), macrophages, vascular endothelial cells (VEC) and alveolar epithelial cells are involved. The PMN, VEC, macrophages and platelets can be activated to produce inflammatory cyto-chemokines such as tumor necrosis factor (TNF-α), interleukin 1β (IL-1β), interleukin 6 (IL-6) and interleukin 8 (IL-8) (Luo et al., 2020). It has been demonstrated that inflammatory cyto-chemokines play important roles in lung injury and the innate immune responses (Baradaran Rahimi et al., 2019). Therefore, regulating exaggerated inflammatory response significantly promotes good prognosis. At present, various drugs with anti-ALI effect, such as dexamethasone, prednisone and ulinastatin, are widely used in the treatment of ALI. However, these drugs exert a variety of side effects, including irregularities of menstruation, hypokalaemia, cyclecoagulation dysfunction, gastric ulcers and osteoporosis, which greatly limited their clinical applications (Mokra et al., 2019). Therefore, it is very important to introduce new technology for ALI therapy with high efficacy, less toxicity and side effects.

In recent years, researchers have been devoted to developing delivery strategies to improve the efficacy of nanotechnology in the treatment of lung diseases. Various nanoparticle-based drug delivery systems, such as polymeric micelles (Wang M. et al., 2020), lipid (de Oliveira et al., 2019), nanocapsules (Chana et al., 2015; Lorenzoni et al., 2019)and nanogel (Ferrer et al., 2014; Merckx et al., 2018) have been used to promote drug delivery to the lungs. For example, Zhang *et al.* developed a new pH-responsive drug delivery system to target inflammatory lungs for ALI therapies. In the ALI mouse model, the nanoparticles can selectively target inflamed endothelium and the lungs after intravenous injection, thus mitigating lung inflammation and injury (Zhang et al., 2019). Kaczmarek reported degradable polymer–lipid nanoparticles with improved serum stability, which was capable of delivering mRNA to the mouse lungs after intravenous administration (Kaczmarek et al., 2016). Using a LPS-induced ALI mouse model, a novel class of peptide-coated gold nanoparticles was able to protect lung from injuries through reducing M2 macrophage polarizations and effectively regulating lung inflammation (Wang L. et al., 2020). A polydopamine nanoparticle was reported acting as a ROS scavenger with remarkable therapeutic effect on acute inflammation-induced injury in both acute peritonitis and ALI murine models (Zhao et al., 2018). Pulmonary drug delivery is a feasible way of drug targeted therapy, due to the surface area of lung epithelium > 100 m^2^ and the thickness of epithelial cell layer < 1 μm, which provides an attractive target for systemic drugs delivery. Nanodrugs could improve drug targeted delivery by achieving a high distributed drug content in alveolus pulmonis (Newman, 2017). Therefore, nanotechnology mediated anti-inflammatory therapy for pulmonary drug delivery is becoming a new strategy for the treatment of ALI.

Artesunate (ART), a water-insoluble derivative of artemisinin, the WHO first-line therapy for most malaria-endemic countries. In addition to its inherent antimalarial activity, ART also exerts various pharmacological activities such as anti-inflammation (Zhang et al., 2020), anticancer (Jiang et al., 2018) and immunomodulation properties (Huang et al., 2019). However, the poor water solubility, short half-life, low bioavailability and growing drug resistance limited the clinical application of artesunate in other indications (Noedl et al., 2008). LPS is one of the major component of cellular membranes of Gram-negative bacteria, which can induce potent inflammatory response leading to lung injury. Multiple studies have demonstrated ART could protect against LPS-induced ALI by inhibiting inflammatory mediator production (Cao et al., 2016; Zhao et al., 2017).

**SCHEME 1:**
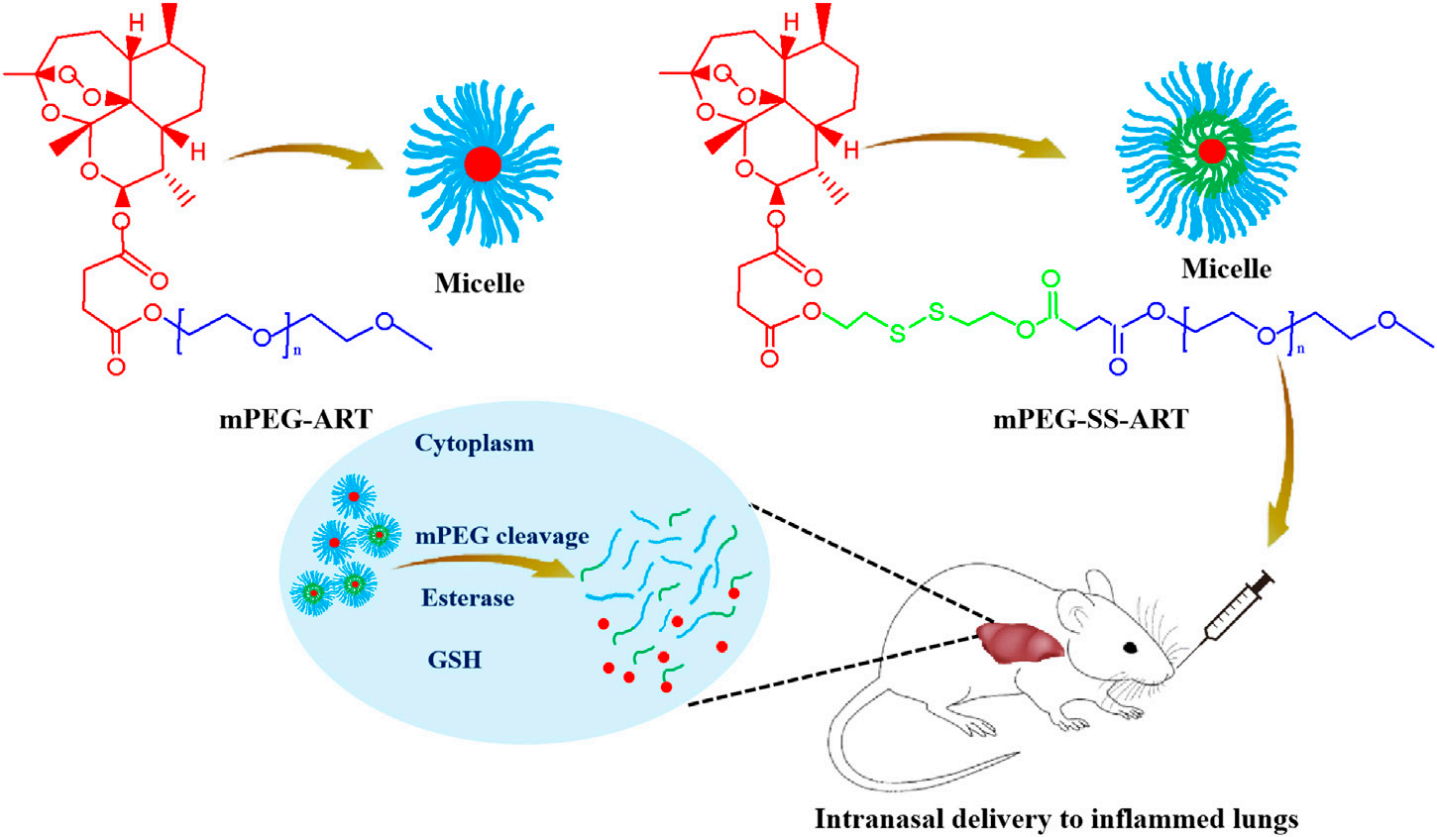
The schematic illustration of the fabrication of the ART-prodrug nanoparticles for improved therapeutic efficacy in the treatment of ALI mice.

Polyethylene glycol (PEG) was widely used as a protective coating on nanoparticles to evade immune clearance and extend the circlation time of drugs. In this study, we designed and synthesized PEGylated ART prodrugs (mPEG-ART) and PEGylated ART prodrugs coupled with disulfide bond (mPEG-SS-ART) (Scheme 1), and assessed whether ART prodrugs could effectively attenuate LPS-induced ALI *in vitro* and *in vivo*. Our results demonstrated the ART prodrugs can effectively alleviate LPS-induced ALI compared with free ART, hinting the potential application of the ART prodrugs for anti-inflammatory purposes.

## Materials and Methods

### Chemical Reagents

Methoxypoly (ethylene glycol) (mPEG, Mw = 2kD and 5kD) was purchased from Sigma-Aldrich (St. Louis, MO, United States). Artesunate (ART) was obtained from Macklin (Shanghai, China). Dihydroartemisinin is a product of Herbest (Baoji, Shaanxi, China). Bis(2-hydroxyethyl) disulfide was provided by Alfa Aesar (Shanghai, China). Succinic anhydride (SA), 4-dimethylaminopyridine (DMAP) and pyrene were purchased from Aladdin (Shanghai, China). *N,N′*-dicyclohexylcarbodiimide was obtained from Adamas (Shanghai, China). Lipopolysaccharide (LPS) and esterase was purchased from Sigma-Aldrich. 3-(4,5-dimethylthiazol-2-yl)-2,5-diphenyltetrazolium bromide (MTT), fetal bovine serum (FBS), dulbecco’s modified eagle medium (DMEM) and trypsin-EDTA were obtained from Gibco (Langley, OK, United States). Tumor necrosis factor alpha (TNF-α), interleukin-6 (IL-6) and interleukin-1β (IL-1β) were obtained from R&D systems (Minneapolis, MN, United States). Myeloperoxidase (MPO) was purchased from Lianke-Bio (Hangzhou, Zhejiang, China). All other chemicals were of analytical or HPLC grade and were used as received.

### Polymer Prodrugs Synthesis and Characterization

mPEG_2k_-ART and mPEG_5k_-ART were synthesized by coupling of the carboxyl group of ART and the hydroxyl group of mPEG_2k_ or mPEG_5k_, respectively. Briefly, 1 mM of mPEG_2k_ or mPEG_5k_ was reacted with an excess of ART by activation with an excess of dicyclohexylcarbodiimide (DCC) and 4-dimethylaminopyridine (DMAP) in 10 ml dichloromethane (DCM) and allowed to react at RT for 24 h. The reaction solution was filtrated through 0.22 μm filter to remove the precipitates. The solvent in the resultant solution was then removed by rotary evaporation at 50°C. The gel like product was dissolved in acetone and subjected to a dialysis bag (MWCO 1000) for purification against DI water for 48 h, and was lyophilized.

mPEG_2k_-SS-ART and mPEG_5k_-SS-ART were prepared through the following procedure. Firstly, 1 mM of mPEG_2k_ and excess of SA were activated by DCC/DMAP following the similar protocol as aforementioned to obtain carboxyl group terminated mPEG_2k_-COOH. Then the product was purified using dialysis bag (MWCO 1000) in DI water for 48 h and was lyophilized. Secondly, 1 mM ART and excess of bis(2-hydroxyethyl) disulfide (HEDS) were activated by excess of DCC/DMAP in 10 ml DCM at RT for 24 h. The solution was filtrated and the filtrate was reacted with activated mPEG_2k_-COOH under the excess amount of DCC/DMAP at RT for24 h mPEG_2k_-SS-ART was obtained by purification as depicted as aforementioned. The synthesis of mPEG_5k_-SS-ART was followed with a similar protocol as that of mPEG_2k_-SS-ART.

^1^H NMR spectra of polymers were obtained by a BrukerAscend^TM^ 600 MHz NMR spectrometer (Billerica, MA, United States) in CDCl_3_ at 25°C.

The drug loading content (LC) was determined by Agilent 1,260 high performance liquid chromatography (HPLC, Santa Clara, CA, United States) equipped with an Agilent C_18_ column (4.6 × 250 mm, 5 μm, column temperature 30°C), with a mobile phase of acetonitrile/phosphoric acid solution (pH 3.0) (52:48, v/v) and a flow rate of 1.0 ml/min. A weighed number of lyophilized micelles was dissolved in methanol and subjected to HPLC detection via a UV detector at 210 nm, as compared to a calibration curve of ART in the mobile phase. The LC was calculated according to the following equation:

$$LC=\frac{weight\;of\;drug\;in\;the\;polymer}{weight\;of\;polymer\;prodrug}\times 100\%$$

### Preparation and Characterization of Polymer Prodrug Micelles

The as-prepared polymer prodrugs could be self-assembled into polymer micelles in aqueous milieu. The polymer micelles were prepared by the thin-film hydration according to previous protocol (Chen et al., 2017).

The critical micelle concentration (CMC) of the polymer prodrugs was determined by fluorescence spectroscopy using pyrene as a probe (Chen et al., 2017). The emission wavelength was set at 390 nm with excitation slit and emission slit of 1.0 and 10 nm, respectively. The excitation intensities at wavelength range from 300 to 360 nm were measured using a HITACHI fluorescence spectrophotometer (F-2k, Tokyo, Japan). The concentration of the polymers solution was varied from 1.0 × 10^−5^ to 1.0 mg/ml containing 6 × 10^−8^ M of pyrene. The fluorescence intensity ratio of I_336_/I_334_ was calculated against polymer concentration to determine CMC. The size and size distribution of the micelles were measured with a Malvern Zetasizer (Nano ZS90, Worcestershire, United Kingdom) with a detection angle at 90°. The stability of the as-prepared polymer micelles was also monitored on the Zetasizer under physiological conditions at 25 and 37°C, respectively. The morphology and structure of the polymer micelles were observed using a HITACHI H7650 (Tokyo, Japan) transmission electron microscope operated at an acceleration voltage of 80 kV. The samples were prepared by dipping 0.1 wt% sample solution onto TEM grids and air dried.

### *In Vitro* Drug Release

The release of ART from the prodrugs was characterized according to our previous study (Chen et al., 2017). In short, a fixed amount of mPEG_2k_-ART or mPEG_5k_-ART micelle (with an ART dosage of 2 mg) was reconstituted in 2 ml PBS with or without the addition of esterase, and was sealed in a dialysis tubing (MWCO 1000) and incubated in 20 ml PBS at pH 7.4. The drug release was carried out at 37°C in an incubator shaking horizontally at 100 rpm. The release medium was completely removed and supplemented with fresh PBS at predetermined time intervals. The ART concentration in the medium was recorded with HPLC with a UV detector operated at 210 nm. The cumulative released drug was integrated from each measurement.

The release of ART from mPEG_2k_-SS-ART or mPEG_5k_-SS-ART prodrugs was same to that of mPEG_2k_-ART except that the esterase was replaced by 10 mM glutathione (GSH).

### Cell Culture

Mouse RAW264.7 cells (the Cell Resource Center of the Institute of Basic Medical Sciences, Chinese Academy of Medical Sciences, Beijing, China) were used and maintained in DMEM supplemented with 10% fetal bovine serum (FBS) and 1% penicillin–streptomycin at 37°C, 5% CO_2_ and 95% humidity.

### Enzyme Linked Immunosorbent Assay

RAW264.7 cells were cultured in DMEM supplemented with 10% fetal bovine serum and 1% antibiotics (100 U/mL penicilin and 100 μg/ml streptomycin, Life Technologies) at 37°C with 5% CO_2_. Cells were seeded in 24-well plates with a density of 2 × 10^5^/well and were treated with PEGylated prodrugs at various ART concentrations (0, 1, 5, 10, 20, 50 μg/ml). 1 h after treatment the cells were challenged with LPS (200 ng/ml) for 6 h. Cell culture supernatants were collected and centrifuged at 10,000 rpm for 10 min at 4°C, then subjected to ELISA of cells secreted TNF-α, IL-1β, IL-6 and total GSH, according to the manufacturer’s instructions (Multisciences Biotech, Hangzhou, China).

### Animals

Kunming mice (male, 4–5 weeks, Beijing Vital River Laboratory Animal Technology Co., Ltd., Beijing, China) were bred in specific pathogen free (SPF) environment. Food and water were available ad libitum. Animal studies were conducted according to the Regulation on Experimental Animals of China Academy of Chinese Medical Sciences.

### *In Vivo* Pulmonary Anti-Inflamation

LPS-induced lung inflammation was investigated on Kunming mice according to protocols reported previously (Ferretti et al., 2003; Bohr et al., 2017). 35 mice were randomly divided into five groups, seven mice per group. Mice were dosed intranasally with 10 mg/kg of ART, mPEG_2k_-ART, mPEG_5k_-ART, mPEG_2k_-SS-ART, mPEG_2k_-SS-ART (1 µL drug solution/g of mice weight), or saline alone, respectively. 1 h later, the mice were nasal administered with LPS solution (1.25 mg/kg) using a pipet by pacing the mice on their backs and allowing them for inhalation of the solution through the nostrils (1 µL LPS solution/g of mice weight). 0.9% NaCl solution was administered in the saline group. The mice were euthanized 6 h later via an over dose of pentobarbital (i.p. injection of 200 μL of pentobarbital solution, 20 mg/ml) for bronchoalveolar lavage (BAL), and the trachea was exposed from an incision and cannulated with a 22-gauge catheter (BD biosciences). Subsequently, BAL was performed by flushing the lungs with 1.0 ml PBS for 2 times and was used for protein quantification and cell counting/differentiation. The BAL was centrifuged at 2 k rpm for 10 min at 4°C, the supernatant was stored at −80°C for protein quantification, and the pellets were pooled and resuspended in fresh PBS.

### Lung wet/dry Ratio

The water content of the lungs was measured following the administration of LPS. The right lungs were excised, blotted, and weighed to obtain the wet weight, then desiccated at 80°C for 24 h to obtain the dry weight. The wet/dry weight ratio was calculated as an assessment of tissue edema.

### Lung Histology

The lung tissues were collected and fixed in 4% paraformaldehyde fixing solution. The tissues were dehydrated, embedded in paraffin, sliced into 5 μm sections. The sections were stained with hematoxylin and eosin (H&E) reagent and visualized under a light microscope.

### Myeloperoxidase Activity Assay

Lung tissues were harvested, rinsed, homogenized, and centrifuged, then the supernatants were collected and subjected to ELISA for determination of myeloperoxidase (MPO) activity using the MPO activity colorimetric assay kit (Biovision, Zurich, Switzerland).

### Neutrophils Assay

The number of neutrophils in each lavage was counted by Wright’s staining using hemocytometer slides. Bronchoalveolar lavage fluid (BALF) samples were collected by flashing the lung three times with 4 ml PBS through a tracheal cannula placed into each mouse under anesthesia. Briefly, animals were sacrificed and the chest was opened, a median sternotomy was performed, and the trachea was isolated using a blunt dissection. Next, a suitable small-caliber tube was inserted into the airway and secured. Then PBS solution was infused slowly into the lungs, and the BALF was withdrawn into the tube. The fluid recovery rate was > 80%. The lavage samples were centrifuged at 1,500 g for 10 min at 4°C.

### Protein Quantification

The protein levels of IL-6, IL-1β, and TNF-α in the BAL were quantified using ELISA. BAL supernatants were stored at −20°C and were used directly or diluted (1:3, v/v) in assay diluent from the kit, and the samples were prepared following the instructions illustrated in the protocol of the kit. All samples were measured in triplicate.

The concentration of TNF-α, IL-1β, and IL-6 in BALF was measured using a commercial ELISA kit. The results were expressed as pg/mL of BALF.

### GSH Measurements

The lung tissue of mice was quickly frozen with liquid nitrogen and ground into powder.The total GSH content of BALF and lung tissue from mice in different treatment groups were determined as the same as the aforementioned.

### Statistical Analysis

Data were processed using GraphPad Prism 5.01 software (La Jolla, CA, United States) and presented as mean ± standard deviation (SD). Statistical analysis was performed using one-way analysis of variance (ANOVA). Tukey’s post-hoc comparisons were used for statistical comparisons. The difference was regarded statistically significant as ^#^ *p* < 0.05 and ^*^ *p* < 0.05, very significant as ^##^ *p* < 0.01, and extremely significant as ^###^ *p* < 0.001.

## Results

### Synthesis and Characterization of PEGylated Art Prodrugs

The poor solubility severely limits the bioavailability of ART both *in vitro* and *in vivo*. At present, special agents or solvents such as NaHCO_3_ have been used to prepare well-dissolved ART stock solutions on account of their poor solubility but shows low bioavailability. The significant effect of PEG-modification may pave new roads in tackling these problems (Turecek et al., 2016). The use of reduction-sensitive disulfide bond for GSH mediated intracellular release has already been studied (Giri et al., 2005) and proved (Wang et al., 2011) to be very effective in reducing off-targeting effect. In this scenario, mPEG_2k_-ART and mPEG_5k_-ART were synthesized by coupling of the carboxyl group of ART and the hydroxyl group of mPEG_2k_ and mPEG_5k_, using dicyclohexylcarbodiimide and 4-dimethylaminopyridine (DCC/DMAP) as catalysts to facilitate the synthesis of ART prodrugs and achieve desirable drug grafting ratio, respectively (Supplementary Figure S1A). mPEG_2k_-SS-ART and mPEG_5k_-SS-ART were synthesized by conjugation of ART with mPEG_2k_ or mPEG_5k_ by coupling with a disulfide bond containing molecule using DCC/DMAP linkage chemistry via the formation of ester bond, respectively (Supplementary Figure S1B). The molar ratio of ART to total matrices was set as a minimum of 3.5:1 (mol/mol) to ensure the successful ligation of one ART molecule to one mPEG chain (Supplementary Table S1). The chemical structures of the prodrugs were characterized by ^1^H NMR spectroscopy, the appearance of peaks at 5.4 ppm (a, 1H), 5.8 ppm (b, 1H) and 2.8 ppm (g, S-CH_2_-CH_2_-), 4.4 ppm (f, S-CH_2_-CH_2_-) were attributed to ART and HEDS, respectively. Particularly, a prominent single peak at 3.4 ppm (e, -OCH_3_) appeared in the spectrum of prodrugs, demonstrating the conjugation of ART and HEDS with mPEG, respectively. The characterization with ^1^ H NMR spectroscopy revealed the ART prodrugs were successfully synthesized (Supplementary Figure S2). The hydrophobic core composed of ART was stabilized by the cloaking mPEG chains, conferring “stealth” properties to the nanosystems in the blood against clearance by the mononuclear phagocyte system for prolonged circulation (Suk et al., 2016). Noted that the inflamed tissues are typically associated with increased reductive and oxidative stress (García et al., 2017). Therefore, the release of ART from mPEG_2k_-SS-ART or mPEG_5k_-SS-ART prodrugs could be mediated by inflammation-associated reductive microenvironment. It is anticipated to realize both prolonged systemic circulation and on-demand drug release, therefore can improve the anti-inflammatory purposes.

The self-assembly behavior of the ART prodrugs in aqueous milieu was evaluated by pyrene fluorescence spectrometry to analyze the critical micelle concentration (CMC) of the prodrugs, due to its high sensitivity to the surrounding polar microenvironment. The intensity ratio (I336/I334) was monitored as a function of ART prodrugs concentration. The intensity ratio (I336/I334) exhibited significant increase with the increase of the prodrug concentration, corresponding to the formation of aggregates together with the accumulation of pyrene into hydrophobic domains. It should be noted that the as-prepared prodrugs exhibited very low CMC values. The CMC values of mPEG_2k_-SS-ART and mPEG_5k_-SS-ART were 0.072 and 0.256 mg mL^−1^, respectively (Supplementary Figure S3). However, the CMC values of mPEG_2k_-ART and mPEG_5k_-ART were not obtained from pyrene fluorescence spectrometry. The results suggested the self-assembly ability of the prodrugs in aqueous medium at low copolymer concentrations. The hydrodynamic diameter of the particles was monitored using dynamic light scattering (DLS). The diameters of ART prodrugs with ester linkages were fairly small with 3.72 and 5.18 nm for mPEG_2k_-ART and mPEG_5k_-ART, respectively (Supplementary Table S2). However, the diameters of mPEG_2k_-SS-ART and mPEG_5k_-SS-ART increased significantly after disulfide bond conjugation, with diameters of 41.04 and 106.35 nm, respectively. The influence of the physiological environment on the size of the prodrug micelles was investigated in PBS (pH 7.4), it can be observed that the sizes of mPEG_2k_-SS-ART and mPEG_5k_-SS-ART micelles decreased significantly in PBS (Supplementary Figure S4), indicating the electrostatic screening effect on the backbone of the prodrugs, leading to more coiled molecular structure in PBS solution. As a comparison, the diameter of mPEG_2k_-ART and mPEG_5k_-ART prodrugs changed slightly after replacement of the medium from DI water to PBS. Transmission electron microscopy (TEM) revealed the spherical structure of the as-prepared micelles, which was in consistence with the results observed using DLS (Supplementary Figure S5).

The stability of the ART prodrugs was investigated by measurement of the micelles’ sizes during incubation at 25 and 37°C according to our previous protocol (Hao et al., 2020). As shown in (Supplementary Figure S6), the mPEG_2k_-ART and mPEG_5k_-ART micelles showed high stability in hydrodynamic diameter within 48 h. However, significant increase in the size of prodrugs was observed after elongation of incubation to 72 h at 25°C, demonstrating the formation of large aggregates. The mPEG_2k_-SS-ART and mPEG_5k_-SS-ART micelles were stable at least within 12 h at both 25 and 37°C, respectively. However, the sizes of the two prodrugs increased significantly after 12 h incubation. The differences in the stability of the prodrugs may be ascribed to the different speed of degradation of ester linkage and disulfide bond (Qiao et al., 2011).

The ester bond can be degraded by esterase, which is abundant in inflammatory tissues (Xu et al., 2008). The nanoparticle carriers stably constrain their drug payloads during systemic circulation but perform esterase-triggered rapid release of drugs in inflammatory tissues (Wang et al., 2021). Dihydroartemisinin (DHA) is an active metabolite of ART. The ART loading content (LC) of the as-prepared prodrugs could be determined by measurement of the DHA content in the presence of porcine liver esterase. As shown in Supplementary Table S1, the LC of ART prodrugs were ranged from 1.4 to 5.7% as determined by HPLC, respectively, which was not in consistence with the result calculated from the ^1^H NMR data, hinting the incomplete dissociation of ART from the respective prodrugs in the current incubation milieu. Therefore, longer incubation time or more esterase would be needed to completely degrade the prodrugs for ART release. However, the solubility of ART was greatly enhanced by the current mPEG modification strategy.

The sustained drug release profile from therapeutic nanoparticles after intravenous administration is particularly important for improving the pharmacokinetics and biodistribution of therapeutic agent (Attia et al., 2013). The *in vitro* release study is important to evaluate whether or not that the prodrugs could release their payload after taken up into cells (Qiao et al., 2021). Considering the formation of drug conjugates via an ester bond or disulfide bond, in this study, the release behavior of ART from the prodrugs was investigated via dialysis method in PBS at 37°C (pH 7.4, with or without esterase for mPEG-ART, with or without GSH for mPEG-SS-ART) to mimic the *in vivo* physiological conditions of blood and cells, respectively. As shown in Supplementary Figure S7, a burst release was observed from mPEG_2k_-ART in PBS with esterase in 8 h and approximately 75% of the drug released. As a comparison, only 27.1% of drug was released from mPEG_2k_-ART in PBS without esterase even after 24 h of incubation, indicating the accelerated drug release profile in the assistance of esterase. However, similar release profiles were observed for mPEG_5k_-ART in PBS with or without esterase, with cumulative drug released amount of 55.6 and 52.1%, respectively. This can be explained that the more coiled structure of mPEG_5k_-ART may impede the access of esterase to the ester bond between mPEG and ART. Subsequently, the drug release profiles of the redox-responsive prodrugs of mPEG_2k_-SS-ART and mPEG_5k_-SS-ART were also investigated. A burst release profile was observed for mPEG_2k_-SS-ART in PBS with the existence of GSH after incubation for 8 h with 50.7% of drug was released, due to the disintegration of disulfide bond under GSH. In comparison, only 26.8% of drug was released from mPEG_2k_-SS-ART in PBS without GSH even after 24 h of incubation. As a contrast, similar release profiles of mPEG_5k_-SS-ART were observed in PBS with or without the addition of GSH, with cumulative drug released amount of 47.2 and 34.3%, respectively, which should be ascribed to the more entangled molecular structure of mPEG_5k_.

**FIGURE 1:**
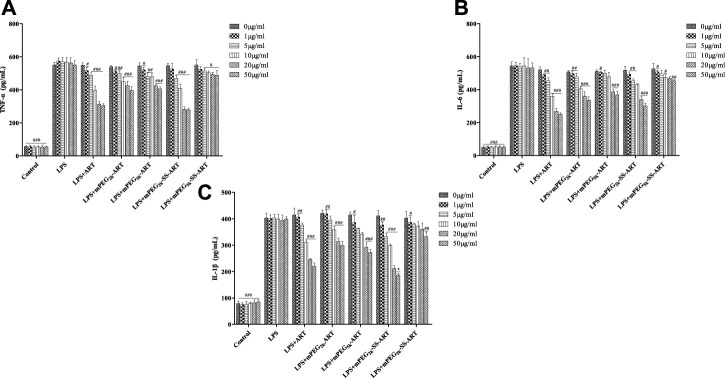
The effect of ART-prodrug treatment on the expression of TNF-α **(A)**, IL-6 **(B)** and IL-1β **(C)** in LPS stimulated RAW264.7 cells. The values were presented as mean ± SD from three independent experiments (*n* = 3). At the same concentration, *p* ^#^<0.05, *p* ^##^<0.01, *p* ^###^<0.001 vs. LPS group, *p* ^*^<0.05 vs ART group, respectively.

ART prodrugs downregulate LPS-induced inflammatory mediators in RAW264.7 cells. The release of inflammatory cytokines plays an essential role in inflammatory process, therefore, the mediation effect of ART prodrugs on the released cytokines from macrophages was investigated. RAW264.7 cells were pretreated with various concentrations of ART prodrugs for 1 h, and stimulated with LPS for 6 h, after which the secretion of TNF-α, IL-1β, and IL-6 in the culture medium was analyzed by ELISA. A significant increase of intracellular inflammatory cytokine levels was observed in the macrophages after stimulation with 200 ng/ml of LPS, as compared to that of the non-stimulated cells. The cytokine levels decreased significantly after treatment by free ART, demonstrating the anti-inflammatory effect of the free drug (Figure 1). Significant downregulation of the cytokines such as TNF-α, IL-6 and IL-1β was observed in the cells treated by mPEG_2k_-ART, mPEG_5k_-ARTand mPEG_2k_-SS-ART, respectively. However, mPEG_5k_-SS-ART showed negligible anti-inflammatory effect at all concentrations investigated. The TNF-α expression can be significantly downregulated by ART and mPEG_2k_-SS-ART at 5 μg mL^−1^ (*p* <0.001) (Figure 1A), with 0.86 and 0.82 fold decrease, respectively. The ART, mPEG_2k_-ART and mPEG_2k_-SS-ART groups showed potent inhibitory effect on IL-6 even at 1 μg mL^−1^ (*p* <0.01) (decreased by 0.90, 0.91, and 0.90 fold, respectively). mPEG_5k_-ART and mPEG_5k_-SS-ART exhibited slight inhibition on IL-6 expression at 20 μg mL^−1^ and 50 μg mL^−1^ (*p* <0.001, *p* <0.01) (Figure 1B). For IL-1β mediation, mPEG_2k_-SS-ART showed significant modulation effect as compared to free ART, whereas both ART and mPEG_2k_-SS-ART exhibited potent inhibitory effects at some concentrations (10 μg mL^−1^, 5 μg mL^−1^, *p* <0.001). However, mPEG_5k_-SS-ART exhibited inhibitory effects only at higher concentrations (50 μg mL^−1^, *p* <0.05) (Figure 1C). The present study showed that the cytokine levels were downregulated more efficiently by the mPEG_2k_-SS-ART than that of the free ART and other ART prodrugs, hinting the inflammation can be effectively inhibited by mPEG_2k_-SS-ARTprodrug at concentrations as low as 10 μg mL^−1^.

**FIGURE 2:**
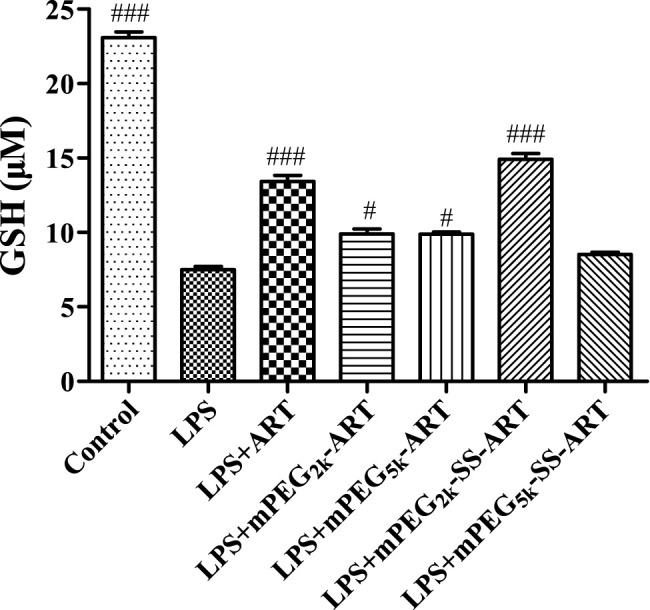
The GSH expression in LPS challenged RAW264.7 cells after intervention by ART prodrugs. The values presented are the means ± SD of three independent experiments (*n* = 3). *p* ^#^<0.05, *p* ^###^<0.001 vs LPS group, respectively.

ART prodrugs protect cells by elevation of intracellular GSH level. Glutathione (GSH) is the most abundant non-protein thiol that defends against oxidative stress but suffers depletion during the inflammatory responses. As shown in Figure 2, the intracellular GSH level was significantly decreased upon LPS stimulation, but that decrease was significantly attenuated in groups co-treated with ART or mPEG_2k_-SS-ART. GSH levels in groups co-treated with ART and mPEG_2k_-SS-ART were 1.79 and 1.99 fold increased compared with LPS group, respectively (at 20 μg mL^−1^). However, no significant upregulation effect on GSH level was observed in the groups of mPEG_2k_-ART, mPEG_5k_-ART and mPEG_5k_-SS-ART, respectively, which might be ascribed to the steric hindrance of mPEG chains which hinders the cellular uptake (Męczyńska-Wielgosz et al., 2016). Our results demonstrated that ART and mPEG_2k_-SS-ART can effectively elicit compensation to oxidative stress by upregulation of GSH levels.

### *In Vivo* Pulmonary Inflamation Inhibition

**FIGURE 3:**
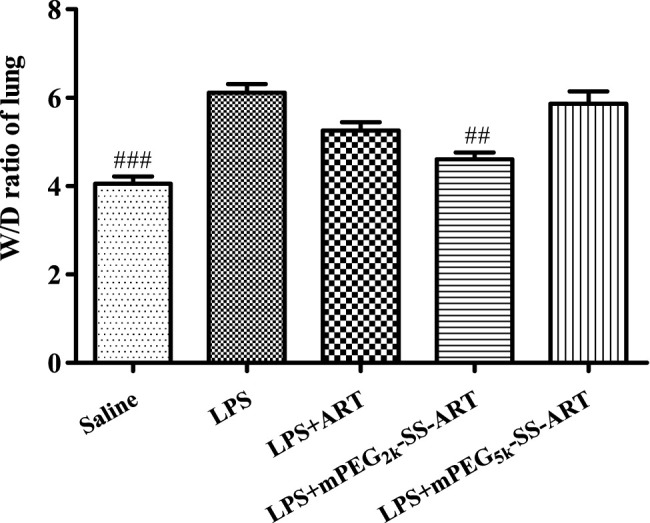
The lung W/D ratio of LPS-induced ALI mice after treatment by ART prodrugs. The values were presented as mean ± SD from three independent experiments (*n* = 3)., *p* ^##^<0.01, *p* ^###^<0.001 vs. LPS group, respectively.

For ALI treatment, nanodrugs are mainly administrated through intrapulmonary or the intravenous route. For the intrapulmonary route, the increase of lung permeability in the pathological environment of ALI may contribute to nanodrug-mediated passive targeted delivery (Newman, 2017). Nanodrugs are easy to penetrate the mucus layer and pass through the cell membrane (Lin and Dean, 2011). Therefore, the anti-inflammatory effect of the as-prepared ART prodrugs was investigated on LPS-induced ALI model via nasal administration for effective delivery to lung, as compared to free ART. Significant pulmonary edema was observed in mice after stimulation with LPS. The wet to dry (W/D) ratio of the lungs in all treatment groups were measured 6 h after LPS challenge. Compared with the saline group, lung W/D ratio was significantly increased in the LPS-treated group. The W/D ratio of the mice was significantly decreased in mPEG_2k_-SS-ARTgroup, as compared to that of the LPS group (Figure 3) (*p* <0.01). It was shown that stimulation of the lung epithelial cells by LPS could induce the release of chemokines and cytokines (Welbourn and Young, 1992). These cytokines could lead to the accumulation of numerous neutrophils into the lung tissues and lead to lung edema. In addition, the overproduction of these inflammatory cytokines leads to the injury of the lung tissues.

**FIGURE 4:**
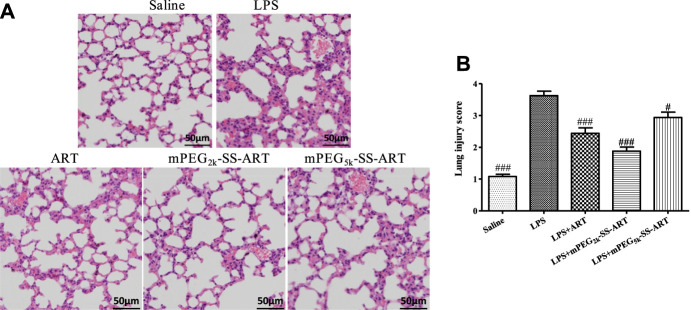
The histopathological changes in lung tissues of LPS-induced ALI mice after treatment by various ART formulations **(A)**, the lung injury scores of the lung tissues **(B)**. Representative histological changes of lung obtained from mice of different groups (*n* = 3). *p* ^#^<0.05, *p* ^###^<0.001 vs. LPS group, respectively.

To further evaluate the anti-inflammatory effect of ART prodrugs, immunohistochemical analysis was performed to evaluate the effect of ART formulations on the LPS-induced histopathological changes. As shown in Figure 4, the structure of normal lung tissues was exhibited in the saline group. Significant pathological changes were observed in the lung tissues of LPS group, including neutrophils infiltration, edema, significant thickening of alveolar walls and alveolar disarray. However, the histopathological deterioration was significantly alleviated after treatment by ART, mPEG_2k_-SS-ART and mPEG_5k_-SS-ART, respectively. And the most potent therapeutic effect was observed in mPEG_2k_-SS-ART group.

**FIGURE 5:**
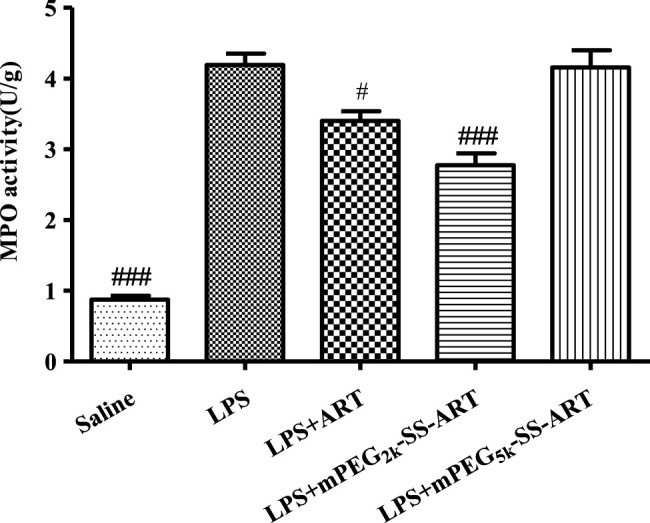
The MPO activity in lung tissues of LPS induced ALI model after treatment by various ART prodrugs. The values presented are mean ± SD of three independent experiments (*n* = 3). *p* ^#^<0.05, *p* ^###^<0.001 vs. LPS group, respectively.

MPO was abnormally produced in neutrophils under inflammatory conditions (Ndrepepa, 2019), therefore the MPO activity in lung was spectrophotometrically assayed using test kits. The lung neutrophil penetration marker of MPO activity was detected 6 h later after LPS stimulation. As illustrated in Figure 5, the lung MPO activity was significantly increased in the LPS group compared with the saline group. However, the MPO activity was dramatically inhibited by free ART and mPEG_2k_-SS-ART, with better therapeutic effect for the mPEG_2k_-SS-ART group. However, negligible inhibitory effect on MPO activity was observed in mPEG_5k_-SS-ART group, which might due to the impedance of longer mPEG chain on the release of the drug (Sanchez et al., 2017).

**FIGURE 6:**
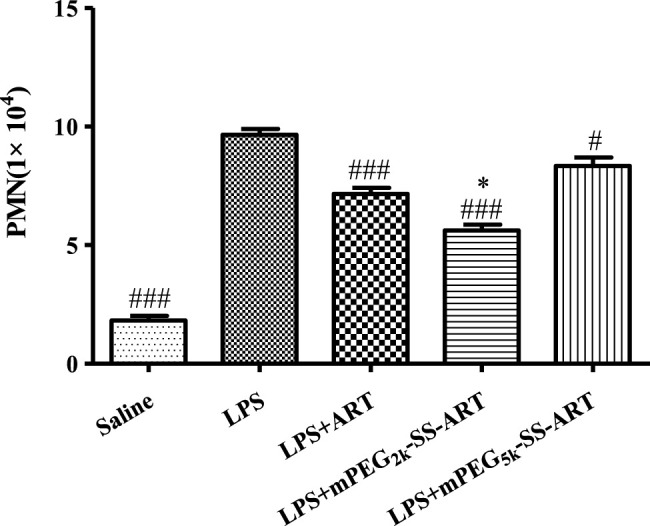
The inflammatory cell infiltration in the BALF solution of LPS-induced ALI mice after treatment by various ART formulations. The values were presented as mean ± SD of three independent experiments (*n* = 3). *p* ^#^<0.05, *p* ^###^<0.001 vs. LPS group, *p* ^*^<0.05 vs. ART group, respectively.

The pathogenesis of ALI was also associated with the vasculature inflammation and neutrophil-mediated inflammation (Thompson et al., 2017). The severity and development of ARDS/ALI was manipulated by the migration of neutrophil into the lungs in response to activated macrophages (Williams and Chambers, 2014). In the current study, the total neutrophil counts of BALF were all significantly down-regulated, suggesting the remarkable therapeutic effect of ART preparations to combat ALI. And best therapeutic efficacy was observed in the mPEG_2k_-SS-ART group, as compared to that of free ART (Figure 6), which might be ascribed to the accelerated phagocytosis by macrophages (Kim et al., 2018), and enhanced neutrophil uptake for smaller sized nanoparticles with reduced cell viability (Yu et al., 2020).

**FIGURE 7:**
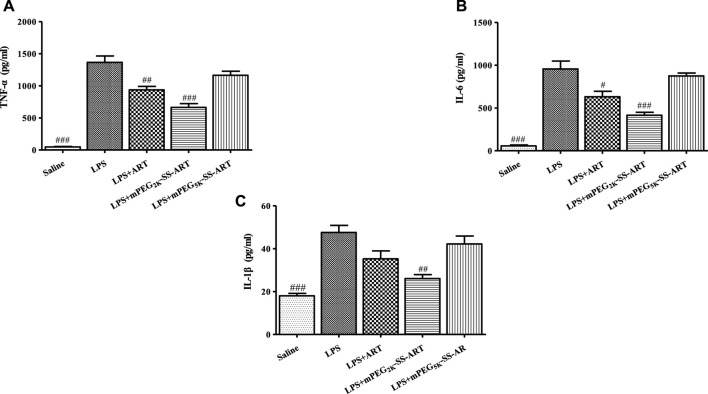
The production of TNF-α **(A)**, IL-6 **(B)** and IL-1β **(C)** in the BALF of LPS-induced ALI mice after treatment by various ART prodrugs. The values were presented as mean ± SD of three independent experiments (*n* = 3). *p* ^#^<0.05, *p* ^##^<0.01, *p* ^###^<0.001 vs. LPS group, respectively.

The inflammatory cytokines played an important role in the development of lung injury (Kim et al., 2019). The inflammation associated cytokines including TNF-α, IL-6 and IL-1β were detected in the BALF of ALI mice by ELISA to investigate the anti-inflammatory effects of ART prodrugs *in vivo*. It has been shown that the level of proinflammatory cytokines such as TNF-α, IL-6 and IL-1β in the lung was significantly increased after LPS stimulation on mice, which is consistent with the anti-inflammatory properties of ART prodrugs *in vitro.* It can be seen that the BALF levels of TNF-α, IL-6 were significantly reduced in the “LPS + ART” group as compared to the LPS group. Moreover, the BALF levels of TNF-α, IL-6 and IL-1β all exhibited significant decrease post mPEG_2k_-SS-ART treatment, demonstrating the better anti-inflammatory effect of mPEG_2k_-SS-ART prodrug in *vivo* studies (Figure 7).

**FIGURE 8:**
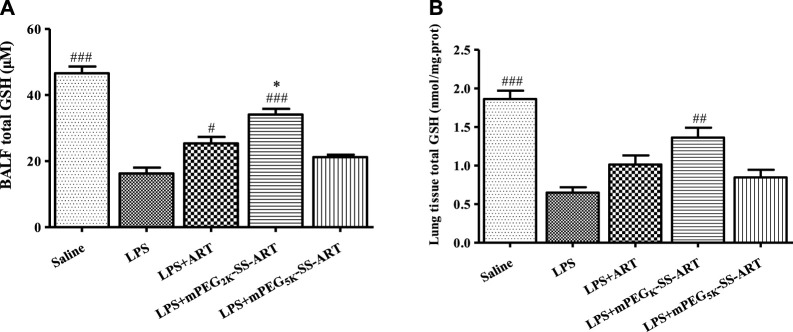
The GSH production in BALF **(A)** and lung tissue **(B)** of LPS-induced ALI mice after treatment by various ART prodrugs. The values presented are mean ± SD of three independent experiments (*n* = 3). *p* ^#^<0.05, *p* ^##^<0.01, *p* ^###^<0.001 vs. LPS group, *p* ^*^<0.05 vs. ART group, respectively.

Furthermore, the levels of GSH in the BALF and the lung tissues were measured in order to explore the possible mode of action of the ART prodrugs. Interestingly, increased GSH levels in the BALF and the lung tissues were observed in free ART and mPEG_2k_-SS-ART group after LPS stimulation. In the mPEG_2k_-SS-ART group, the GSH levels in the BALF and in the lung tissue increased significantly (2.09 and 2.10 fold, respectively). However, in the free ART group, the GSH level only significantly increased in the BALF (1.55 fold) solution. On another hand, mPEG_5k_-SS-ART showed a similar trend of increased GSH level but not statistically significant, with a comparison to the LPS stimulated group. Therefore, it could be indicated that mPEG_2k_-SS-ART may be the best ART prodrugs on account of anti-inflammatory applications (Figure 8).

## Conclusion

In the present study, ART prodrugs were synthesized by mPEG-modification with ester bond or disulfide linkage, which were developed for the treatment of acute inflammation induced lung injury. The anti-inflammatory effect of the ART prodrugs was investigated both *in vitro* and *in vivo*. The inflammatory cytokine levels were significantly down-regulated and the GSH level was dominated up-regulated in the macrophages treated by mPEG_2k_-SS-ART prodrug post inflammatory stimulation. The anti-inflammation activity of ART prodrugs was further evaluated in the ALI model. The animal lung injury was markedly alleviated after treatment by free ART and mPEG_2k_-SS-ART, as observed in the significantly reduced wet to dry lung ratio, improved lung morphological alterations, reduced lung MPO activity, decreased total neutrophil counts of BALF, down-regulated inflammatory cytokine levels of BALF and increased GSH levels. And better therapeutic effect was observed inmPEG_2k_-SS-ART group compared with free ART. Therefore, the mPEG_2k_-SS-ART prodrug may serve as a potential candidate for the treatment of ALI. Still, there are limitations to be settled. The current study emphasized PEGylated ART prodrugs for early lung injury, while in many cases, ALI could develop ARDS and may lead to serious dysfunction of the respiratory system. Therefore, the interruption of the progression from ALI to ARDS should be considered. The overwhelming inflammation and destruction of lung barrier are the fundamental pathophysiology involved in the development of ARDS. Given the complexity of ARDS disease, single-drug therapy may not be enough to blockade the progression of the illness. PEGylated ART prodrugs co-delivery system, that is, targeting multiple inflammatory and pathways simultaneously, may be a future direction to optimize the treatment of ARDS and frustrate the progression of the disease.

## Data Availability

The original contributions presented in the study are included in the article/Supplementary Material, further inquiries can be directed to the corresponding authors.

## References

[B1] Ashbaugh D. G. Bigelow D. B. Petty T. L. Levine B. E. (1967). Acute Respiratory Distress in Adults. Lancet 2, 319–323. 10.1016/s0140-6736(67)90168-7 4143721

[B2] Attia A. B. Yang C. Tan J. P. Gao S. Williams D. F. Hedrick J. L. (2013). The Effect of Kinetic Stability on Biodistribution and Anti-tumor Efficacy of Drug-Loaded Biodegradable Polymeric Micelles. Biomaterials 34, 3132–3140. 10.1016/j.biomaterials.2013.01.042 23380357

[B3] Baradaran rahimi V. Rakhshandeh H. Raucci F. Buono B. Shirazinia R. Samzadeh Kermani A. (2019). Anti-Inflammatory and Anti-oxidant Activity of Portulaca Oleracea Extract on LPS-Induced Rat Lung Injury. Molecules 24. 10.3390/molecules24010139 PMC6337267 30609661

[B4] Bellani G. Laffey J. G. Pham T. Fan E. Brochard L. Esteban A. (2016). Epidemiology, Patterns of Care, and Mortality for Patients with Acute Respiratory Distress Syndrome in Intensive Care Units in 50 Countries. JAMA 315, 788–800. 10.1001/jama.2016.0291 26903337

[B5] Bohr A. Tsapis N. Andreana I. Chamarat A. Foged C. Delomenie C. (2017). Anti-Inflammatory Effect of Anti-TNF-α SiRNA Cationic Phosphorus Dendrimer Nanocomplexes Administered Intranasally in a Murine Acute Lung Injury Model. Biomacromolecules 18, 2379–2388. 10.1021/acs.biomac.7b00572 28639789

[B6] Butt Y. Kurdowska A. Allen T. C. (2016). Acute Lung Injury: A Clinical and Molecular Review. Arch. Pathol. Lab. Med. 140, 345–350. 10.5858/arpa.2015-0519-RA 27028393

[B7] Cao T. H. Jin S. G. Fei D. S. Kang K. Jiang L. Lian Z. Y. (2016). Artesunate Protects against Sepsis-Induced Lung Injury via Heme Oxygenase-1 Modulation. Inflammation 39, 651–662. 10.1007/s10753-015-0290-2 26627481

[B8] Chana J. Forbes B. Jones S. A. (2015). Triggered-release Nanocapsules for Drug Delivery to the Lungs. Nanomedicine 11, 89–97. 10.1016/j.nano.2014.07.012 25101879

[B9] Chen Y. Yue Q. De G. Wang J. Li Z. Xiao S. (2017). Inhibition of Breast Cancer Metastasis by Paclitaxel-Loaded pH Responsive Poly(β-Amino Ester) Copolymer Micelles. Nanomedicine (Lond) 12, 147–164. 10.2217/nnm-2016-0335 27854565

[B10] de Oliveira M. T. P. de Sá Coutinho D. Tenório de Souza É. Stanisçuaski Guterres S. Pohlmann A. R. Silva P. M. R. (2019). Orally Delivered Resveratrol-Loaded Lipid-Core Nanocapsules Ameliorate LPS-Induced Acute Lung Injury via the ERK and PI3K/Akt Pathways. Int. J. Nanomedicine 14, 5215–5228. 10.2147/IJN.S200666 31371957 PMC6636190

[B11] Ferrer M. C. Shuvaev V. V. Zern B. J. Composto R. J. Muzykantov V. R. Eckmann D. M. (2014). Icam-1 Targeted Nanogels Loaded with Dexamethasone Alleviate Pulmonary Inflammation. PLoS One 9, e102329. 10.1371/journal.pone.0102329 25019304 PMC4096597

[B12] Ferretti S. Bonneau O. Dubois G. R. Jones C. E. Trifilieff A. (2003). IL-17, Produced by Lymphocytes and Neutrophils, Is Necessary for Lipopolysaccharide-Induced Airway Neutrophilia: IL-15 as a Possible Trigger. J. Immunol. 170, 2106–2112. 10.4049/jimmunol.170.4.2106 12574382

[B13] Gajic O. Dabbagh O. Park P. K. Adesanya A. Chang S. Y. Hou P. (2011). Early Identification of Patients at Risk of Acute Lung Injury: Evaluation of Lung Injury Prediction Score in a Multicenter Cohort Study. Am. J. Respir. Crit. Care Med. 183, 462–470. 10.1164/rccm.201004-0549OC, 20802164 PMC3056224

[B14] García N. Zazueta C. Aguilera-Aguirre L. (2017). Oxidative Stress and Inflammation in Cardiovascular Disease. Oxid Med. Cel Longev 2017, 5853238. 10.1155/2017/5853238 PMC5426074 28536646

[B15] Giri S. Trewyn B. G. Stellmaker M. P. Lin V. S. (2005). Stimuli-responsive Controlled-Release Delivery System Based on Mesoporous Silica Nanorods Capped with Magnetic Nanoparticles. Angew. Chem. Int. Ed. Engl. 44, 5038–5044. 10.1002/anie.200501819 16038000

[B16] Hao D. L. Xie R. De G. J. Yi H. Zang C. Yang M. Y. (2020). pH-Responsive Artesunate Polymer Prodrugs with Enhanced Ablation Effect on Rodent Xenograft Colon Cancer. Int. J. Nanomedicine 15, 1771–1786. 10.2147/IJN.S242032 32214810 PMC7083641

[B17] He Y. Q. Zhou C. C. Yu L. Y. Wang L. Deng J. L. Tao Y. L. (2021). Natural Product Derived Phytochemicals in Managing Acute Lung Injury by Multiple Mechanisms. Pharmacol. Res. 163, 105224. 10.1016/j.phrs.2020.105224 33007416 PMC7522693

[B18] Huang Z. Z. Xu Y. Xu M. Shi Z. R. Mai S. Z. Guo Z. X. (2019). Artesunate Alleviates Imiquimod-Induced Psoriasis-like Dermatitis in BALB/c Mice. Int. Immunopharmacol 75, 105817. 10.1016/j.intimp.2019.105817 31446161

[B19] Huppert L. A. Matthay M. A. Ware L. B. (2019). Pathogenesis of Acute Respiratory Distress Syndrome. Semin. Respir. Crit. Care Med. 40, 31–39. 10.1055/s-0039-1683996 31060086 PMC7060969

[B20] Jiang F. Zhou J. Y. Zhang D. Liu M. H. Chen Y. G. (2018). Artesunate Induces Apoptosis and Autophagy in HCT116 colon Cancer Cells, and Autophagy Inhibition Enhances the Artesunate-induced A-poptosis. Int. J. Mol. Med. 42, 1295–1304. 10.3892/ijmm.2018.3712 29901098 PMC6089754

[B21] Kaczmarek J. C. Patel A. K. Kauffman K. J. Fenton O. S. Webber M. J. Heartlein M. W. (2016). Polymer-Lipid Nanoparticles for Systemic Delivery of mRNA to the Lungs. Angew. Chem. Int. Ed. Engl. 55, 13808–13812. 10.1002/anie.201608450 27690187 PMC5279893

[B22] Kim G. Piao C. Oh J. Lee M. (2019). Combined Delivery of Curcumin and the Heme Oxygenase-1 Gene Using Cholesterol-Conjugated Polyamidoamine for Anti-inflammatory Therapy in Acute Lung Injury. Phytomedicine 56, 165–174. 10.1016/j.phymed.2018.09.240 30668337

[B23] Kim G. Piao C. Oh J. Lee M. (2018). Self-assembled Polymeric Micelles for Combined Delivery of Anti-inflammatory Gene and Drug to the Lungs by Inhalation. Nanoscale 10, 8503–8514. 10.1039/c8nr00427g 29693671

[B24] Lin X. Dean D. A. (2011). Gene Therapy for ALI/ARDS. Crit. Care Clin. 27, 705–718. 10.1016/j.ccc.2011.04.002 21742224 PMC3482940

[B25] Lorenzoni R. Cordenonsi L. M. Davies S. Antonow M. B. Medina Diedrich A. S. Santos C. G. (2019). Lipid-core Nanocapsules Are an Alternative to the Pulmonary Delivery and to Increase the Stability of Statins. J. Microencapsul 36, 317–326. 10.1080/02652048.2019.1624849 31159613

[B26] Luo Q. Zhu J. Zhang Q. Xie J. Yi C. Li T. (2020). MicroRNA-486-5p Promotes Acute Lung Injury via Inducing Inflammation and Apoptosis by Targeting OTUD7B. Inflammation 43, 975–984. 10.1007/s10753-020-01183-3 31940107

[B27] Matthay M. A. Zemans R. L. Zimmerman G. A. Arabi Y. M. Beitler J. R. Mercat A. (2019). Acute respiratory distress syndrome. Nat Rev Dis Primers *,* 5, 18. 10.1038/s41572-019-0069-0 30872586 PMC6709677

[B28] Męczyńska-Wielgosz S. Piotrowska A. Majkowska-Pilip A. Bilewicz A. Kruszewski M. (2016). Effect of Surface Functionalization on the Cellular Uptake and Toxicity of Nanozeolite A. Nanoscale Res. Lett. 11, 123. 10.1186/s11671-016-1334-8 26935303 PMC4775514

[B29] Merckx P. De Backer L. Van Hoecke L. Guagliardo R. Echaide M. Baatsen P. (2018). Surfactant Protein B (SP-B) Enhances the Cellular siRNA Delivery of Proteolipid Coated Nanogels for Inhalation Therapy. Acta Biomater. 78, 236–246. 10.1016/j.actbio.2018.08.012 30118853

[B30] Mokra D. Mikolka P. Kosutova P. Mokry J. (2019). Corticosteroids in Acute Lung Injury: The Dilemma Continues. Int. J. Mol. Sci. 20. 10.3390/ijms20194765 PMC6801694 31557974

[B31] Ndrepepa G. (2019). Myeloperoxidase - A Bridge Linking Inflammation and Oxidative Stress with Cardiovascular Disease. Clin. Chim. Acta 493, 36–51. 10.1016/j.cca.2019.02.022 30797769

[B32] Newman S. P. (2017). Drug Delivery to the Lungs: Challenges and Opportunities. Ther. Deliv. 8, 647–661. 10.4155/tde-2017-0037 28730933

[B33] Noedl H. Se Y. Schaecher K. Smith B. L. Socheat D. Fukuda M. M. (2008). Evidence of Artemisinin-Resistant Malaria in Western Cambodia. N. Engl. J. Med. 359, 2619–2620. 10.1056/NEJMc0805011 19064625

[B34] Qiao Q. Liu X. Yang T. Cui K. Kong L. Yang C. (2021). Nanomedicine for Acute Respiratory Distress Syndrome: The Latest Application, Targeting Strategy, and Rational Design. Acta Pharm. Sin B 11, 3060–3091. 10.1016/j.apsb.2021.04.023 33977080 PMC8102084

[B35] Qiao Z. Y. Zhang R. Du F. S. Liang D. H. Li Z. C. (2011). Multi-responsive Nanogels Containing Motifs of Ortho Ester, Oligo(ethylene Glycol) and Disulfide Linkage as Carriers of Hydrophobic Anti-cancer Drugs. J. Control. Release 152, 57–66. 10.1016/j.jconrel.2011.02.029 21392550

[B36] Ranieri V. M. Ranieri V. M. Rubenfeld G. D. Thompson B. T. Ferguson N. D. Caldwell E. (2012). Acute Respiratory Distress Syndrome: the Berlin Definition. Jama 307, 2526–2533. 10.1001/jama.2012.5669 22797452

[B37] Rehberg S. Maybauer M. O. Enkhbaatar P. Maybauer D. M. Yamamoto Y. Traber D. L. (2009). Pathophysiology, Management and Treatment of Smoke Inhalation Injury. Expert Rev. Respir. Med. 3, 283–297. 10.1586/ERS.09.21 20161170 PMC2722076

[B38] Ruthman C. A. Festic E. (2015). Emerging Therapies for the Prevention of Acute Respiratory Distress Syndrome. Ther. Adv. Respir. Dis. 9, 173–187. 10.1177/1753465815585716 26002528 PMC4659368

[B39] Sakai L. Gaspar H. A. Ferranti J. F. Carvalho W. B. Delgado A. F. (2014). Acute Respiratory Distress Syndrome in Children: Is There Any Evidence to Use Surfactant? Pediatr. Crit. Care Med. 15, 183–184. 10.1097/PCC.0000000000000026 24492193

[B40] Sanchez L. Yi Y. Yu Y. (2017). Effect of Partial PEGylation on Particle Uptake by Macrophages. Nanoscale 9, 288–297. 10.1039/c6nr07353k 27909711 PMC6397647

[B41] Shaw T. D. Mcauley D. F. O'Kane C. M. (2019). Emerging Drugs for Treating the Acute Respiratory Distress Syndrome. Expert Opin. Emerg. Drugs 24, 29–41. 10.1080/14728214.2019.1591369 30841764

[B42] Suk J. S. Xu Q. Kim N. Hanes J. Ensign L. M. (2016). PEGylation as a Strategy for Improving Nanoparticle-Based Drug and Gene Delivery. Adv. Drug Deliv. Rev. 99, 28–51. 10.1016/j.addr.2015.09.012 26456916 PMC4798869

[B43] Thompson B. T. Chambers R. C. Liu K. D. (2017). Acute Respiratory Distress Syndrome. N. Engl. J. Med. 377, 562–572. 10.1056/NEJMra1608077 28792873

[B44] Turecek P. L. Bossard M. J. Schoetens F. Ivens I. A. (2016). PEGylation of Biopharmaceuticals: A Review of Chemistry and Nonclinical Safety Information of Approved Drugs. J. Pharm. Sci. 105, 460–475. 10.1016/j.xphs.2015.11.015 26869412

[B45] Wang L. Zhang H. Sun L. Gao W. Xiong Y. Ma A. (2020a). Manipulation of Macrophage Polarization by Peptide-Coated Gold Nanoparticles and its Protective Effects on Acute Lung Injury. J. Nanobiotechnology 18, 38. 10.1186/s12951-020-00593-7 32101146 PMC7045427

[B46] Wang M. Wang K. Deng G. Liu X. Wu X. Hu H. (2020b). Mitochondria-Modulating Porous Se@SiO2 Nanoparticles Provide Resistance to Oxidative Injury in Airway Epithelial Cells: Implications for Acute Lung Injury. Int. J. Nanomedicine 15, 2287–2302. 10.2147/IJN.S240301 32280221 PMC7127826

[B47] Wang Y. Xie H. Ying K. Xie B. Chen X. Yang B. (2021). Tuning the Efficacy of Esterase-Activatable Prodrug Nanoparticles for the Treatment of Colorectal Malignancies. Biomaterials 270, 120705. 10.1016/j.biomaterials.2021.120705 33581609

[B48] Wang Y. C. Wang F. Sun T. M. Wang J. (2011). Redox-responsive Nanoparticles from the Single Disulfide Bond-Bridged Block Copolymer as Drug Carriers for Overcoming Multidrug Resistance in Cancer Cells. Bioconjug. Chem. 22, 1939–1945. 10.1021/bc200139n 21866903

[B49] Welbourn C. R. Young Y. (1992). Endotoxin, Septic Shock and Acute Lung Injury: Neutrophils, Macrophages and Inflammatory Mediators. Br. J. Surg. 79, 998–1003. 10.1002/bjs.1800791006 1422741

[B50] Williams A. E. Chambers R. C. (2014). The Mercurial Nature of Neutrophils: Still an enigma in ARDS? Am. J. Physiol. Lung Cel Mol Physiol 306, L217–L230. 10.1152/ajplung.00311.2013 PMC3920201 24318116

[B51] Xu H. Deng Y. Chen D. Hong W. Lu Y. Dong X. (2008). Esterase-catalyzed dePEGylation of pH-Sensitive Vesicles Modified with Cleavable PEG-Lipid Derivatives. J. Control. Release 130, 238–245. 10.1016/j.jconrel.2008.05.009 18657874

[B52] Yu H. P. Liu F. C. Umoro A. Lin Z. C. Elzoghby A. O. Hwang T. L. (2020). Oleic Acid-Based Nanosystems for Mitigating Acute Respiratory Distress Syndrome in Mice through Neutrophil Suppression: How the Particulate Size Affects Therapeutic Efficiency. J. Nanobiotechnology 18, 25. 10.1186/s12951-020-0583-y 32005196 PMC6995149

[B53] Zhang C. Y. Lin W. Gao J. Shi X. Davaritouchaee M. Nielsen A. E. (2019). pH-Responsive Nanoparticles Targeted to Lungs for Improved Therapy of Acute Lung Inflammation/Injury. ACS Appl. Mater. Inter. 11, 16380–16390. 10.1021/acsami.9b04051 PMC6542597 30973702

[B54] Zhang E. Wang J. Chen Q. Wang Z. Li D. Jiang N. (2020). Artesunate Ameliorates Sepsis-Induced Acute Lung Injury by Activating the mTOR/AKT/PI3K axis. Gene 759, 144969. 10.1016/j.gene.2020.144969 32712064

[B55] Zhao D. Zhang J. Xu G. Wang Q. (2017). Artesunate Protects LPS-Induced Acute Lung Injury by Inhibiting TLR4 Expression and Inducing Nrf2 Activation. Inflammation 40, 798–805. 10.1007/s10753-017-0524-6 28315999

[B56] Zhao H. Zeng Z. Liu L. Chen J. Zhou H. Huang L. (2018). Polydopamine Nanoparticles for the Treatment of Acute Inflammation-Induced Injury. Nanoscale 10, 6981–6991. 10.1039/c8nr00838h 29610822

