# Supplementary material for: The Alleviation of LPS-Induced Murine Acute Lung Injury by GSH-Mediated PEGylated Artesunate Prodrugs
Source: Front Pharmacol. 2022 May 20;13:860492. doi: 10.3389/fphar.2022.860492 (PMC9163345; doi:10.3389/fphar.2022.860492)
Supplement: Supplementary file 1 [file DataSheet1.docx]

**Supporting Figure Legends**

**Figure S1.** The schematic illustration of the synthesis of mPEG_2K_-ART, mPEG_5K_-ART (A) and mPEG_2K_-SS-ART, mPEG_5K_-SS-ART (B).

**Table S1.** The drug content of ART in the polymer-ART conjugates micelles as determined by HPLC and ^1^H-NMR, respectively (n=3).

**Figure S2.** The ^1^HNMR spectra of ART, mPEG-ART, mPEG-SS-ART, CDCl_3_ was used as solvent.

**Figure S3.** The critical micelle concentration (CMC) of mPEG_2k_-SS-ART (A) and mPEG_5k_-SS-ART (B), as measured by pyrene fluorescence spectroscopy.

**Table S2.** The size and size distribution of polymer-ART conjugates micelles.

**Figure S4.** The size and size distribution of ART prodrugs in water and PBS (pH 7.4), (A) and (B), respectively.

**Figure S5.** The size and size distribution and transmission electron microscopy (TEM) images of the micelles in PBS (pH 7.4). (A) mPEG_2k_-ART, (B) mPEG_5k_-ART, (C) mPEG_2k_-SS-ART, and (D) mPEG_5k_-SS-ART, respectively.

**Figure S6.** The stability of ART-prodrug micelles incubated in PBS (pH 7.4) at 25 °C (A) and 37 °C (B) for 72 h, (n=3).

**Figure S7.** The *In vitro* drug release profile of ART from the mPEG-ART (A) and mPEG-SS-ART (B) prodrug micelles at various environment (n=3).
